# Analysis of percutaneous kyphoplasty or short-segmental fixation combined with vertebroplasty in the treatment of Kummell disease

**DOI:** 10.1186/s13018-019-1358-8

**Published:** 2019-09-13

**Authors:** Wei Lu, Long Wang, Chunlin Xie, Zhaowei Teng, Gonghai Han, Rongmao Shi, Jinlong Liang, Sheng Lu

**Affiliations:** 1 Clinical College of 920th Hospital of Joint Logistics Support Force of Kunming Medical University, No. 212 Daguan Road, Kunming, 65032 Yunnan People’s Republic of China; 2 Zhengzhou Orthopaedic Hospital, Zhengzhou, 450052 Henan, People’s Republic of China; 3 Department of Thoracic Surgery, The First Affiliated Hospital of Kunming Medical University, Kunming, 650032 Yunnan People’s Republic of China; 4 Department of Orthopedics, The Sixth Affiliated Hospital of Kunming Medical University, Yuxi, 653100 Yunan, People’s Republic of China; 5 Department of Orthopaedics, The First Peoples’ Hospital of Yunnan Province, Kunming, 650032 Yunnan People’s Republic of China; 6 The Affiliated Hospital of Kunming University of Science and Technology, Kunming, 650032 Yunnan People’s Republic of China

**Keywords:** Kyphoplasty, Vertebroplasty, Fixation, Kummell disease

## Abstract

**Background:**

In recent years, short segment internal fixation combined with vertebroplasty (SSF + VP) has provided a new option for the treatment of Kummell disease (KD). The purpose of this study is to evaluate the efficacy of percutaneous kyphoplasty (PKP) and SSF + VP, to provide evidence-based medical support for the decision-making process when treating KD patients without neurological deficits.

**Methods:**

Databases including MEDLINE (PubMed) and EMBASE (Ovid) (1947 to April 6, 2019) were searched for PKP and short-segmental fixation combined with vertebroplasty (SSF + VP) to treat Kummell disease in randomized controlled trials (RCTs) or cohort studies. Two investigators independently evaluated the eligibility of the studies retrieved from the databases based on the predetermined selection criteria. The postoperative evaluation indexes included operation time, bleeding volume, visual analog scale (VAS) score, Oswestry Disability Index (ODI) score, local vertebral Cobb angle, and cement leakage. When the data were significant, a random-effects model was used for analysis. In contrast, when the results showed no statistical heterogeneity, a fixed-effects model was used to estimate the overall effect sizes.

**Results:**

Three retrospective case-control studies were included in the final analysis. The differences in the bleeding volume and operation time were statistically significant, and the combined weighted mean differences (WMDs) (95% CI) were − 0.204.46 (− 210.97, − 197.93) and − 98.98 (− 141.63, 56.32), respectively.

The combined data showed that the differences in VAS score, ODI score, local vertebral Cobb angle, and cement leakage were not statistically significant.

**Conclusions:**

This analysis demonstrates that the PKP and SSF + VP methods are safe and effective in treating Kummell disease patients without neurological symptoms. However, PKP can shorten the operation time and reduce the volume of blood loss.

## Background

Kummell disease (KD) was first reported by Steel in 1951 and occurs in middle-aged and elderly people with osteoporosis. KD presents as a delayed vertebral compression fracture and is characterized by the following common characteristics: a history of minor trauma, after which the pain disappears, but the symptoms recur or worsen, and a kyphosis deformity occurs months or years later [1, 2]. The affected vertebra is usually located in the lower thoracic or upper lumbar region (T8–L4), owing to the well-known prevalence of vertebral fractures at the thoracolumbar junction. In the majority of cases, only a single vertebra is involved [2].

The main diagnostic imaging finding of KD is characterized by an intravertebral vacuum cleft on plain radiograph, which is better appreciated on the anteroposterior view of computed tomography (CT) or magnetic resonance imaging (MRI) scans [3]. However, imaging cannot be used as a specific basis for diagnosis. KD is surrounded by hardened bone and cannot self-heal. At present, there is no standard treatment for KD [1, 4].

In terms of surgical treatments, different methods are adopted according to the presence of neurological symptoms. If the patients are neurologically impaired, the aim of surgery is to decompress the spinal canal, restore the spinal curvature, and maintain spinal stability. The surgical modes include anterior, posterior, or combined anterior and posterior approaches. If the patients do not have neurological symptoms, the purpose of surgery is to preserve the maximum amount of movement of the injured vertebra and to restore vertebral height and sagittal alignment. Therefore, the percutaneous kyphoplasty (PKP) and percutaneous vertebroplasty (PVP) techniques have been widely used to treat KD [5, 6]. However, there have been reports of loosening and displacement of the bone cement, further loss of vertebral height, and even secondary paralysis following PKP or PVP [7–10]. Therefore, short-segmental fixation combined with vertebroplasty (SSF + VP) has also been used to treat KD in recent years. This technique has been reported to have certain positive effects on pain relief and functional recovery [1, 11, 12].

However, no consensus has been reached on the optimal treatment method for KD patients without neurological deficits. Thus, we performed an analysis to evaluate the efficacy of PKP and SSF + VP and to provide evidence-based medical support for the decision-making process when treating KD patients without neurological deficits.

## Methods

### Search strategy and data sources

We searched MEDLINE (PubMed) and EMBASE (OVID) (1947 to April 6, 2019) for randomized controlled trials (RCTs) or cohort studies that investigated PKP and SSF + VP to treat KD. There were no restrictions regarding language or type of publication. The search terms used were the following: (i) kummell [Title/Abstract]) OR avascular osteonecrosis of vertebral body [Title/Abstract]) OR vertebral osteonecrosis [Title/Abstract]) OR vertebral pseudarthrosis [Title/Abstract]) OR intravertebral vacuum cleft [Title/Abstract]) OR delayed vertebral collapse [Title/Abstract]) OR compression fracture nonunion [Title/Abstract]; AND (ii) kyphoplasty [Title/Abstract]) OR vertebroplasty [Title/Abstract]) OR bone cement augment [Title/Abstract]) OR fixation [Title/Abstract]. The retrieval strategy was formulated according to a professional retrieval process, and we also searched the bibliographies of relevant articles to identify any additional studies.

### Study selection

The inclusion criteria were as follows: (1) presented original data from a cohort study or case-control study; (2) included patients definitively diagnosed with KD; (3) used two comparator groups in which one group was treated with a PKP strategy, and the other group was treated with an SSF + VP strategy; (4) included patients with monosegmental lesions who did not have neurological deficits and for whom conservative treatment was invalid; and (5) had sufficient data for analysis.

The exclusion criteria were as follows: (1) included patients with metastatic spinal tumors, infections, primary bone tumors, multiple myeloma or bisegmental and multisegmental lesions; (2) included patients with neurological symptoms; (3) included patients with defects of the posterior wall of the vertebral body or those that occupied the vertebral canal; and (4) included non-human study subjects. If the data were duplicated or the same population was used in more than one study, we chose the most recent or complete study.

### Data extraction and quality assessment

Two investigators (Wei Lu/Zhaowei Teng) independently evaluated the eligibility of the studies retrieved from the databases based on the predetermined selection criteria. In addition, a cross-reference search for eligible articles was conducted to identify studies not identified from the computerized search. These two authors independently extracted the following data: the first author’s name; year of publication, study regions, cohort size, operative time, bleeding volume, visual analog scale (VAS) score, Oswestry Disability Index (ODI) score, local vertebral Cobb angle, cement leakage**,** and statistical adjustments for confounding factors. Any disagreements were resolved either by discussion or in consultation with the corresponding author (Sheng Lu). Finally, the eligible studies were included in the meta-analysis.

### Statistical analyses

Data analysis was performed using Stata 14.0 software (StataCorp., USA). The Cochran *Q* and *I*^2^ value were used together to test heterogeneity. When the *p* value was < 0.1 and the *I*^2^ value was > 50%, the data were considered to be heterogeneous, and a random-effects model was used for the meta-analysis. Otherwise, when the results showed no statistical heterogeneity, a fixed-effects model was used to estimate the overall effect sizes.

## Results

### Literature search and study characteristics

**Fig. 1:**
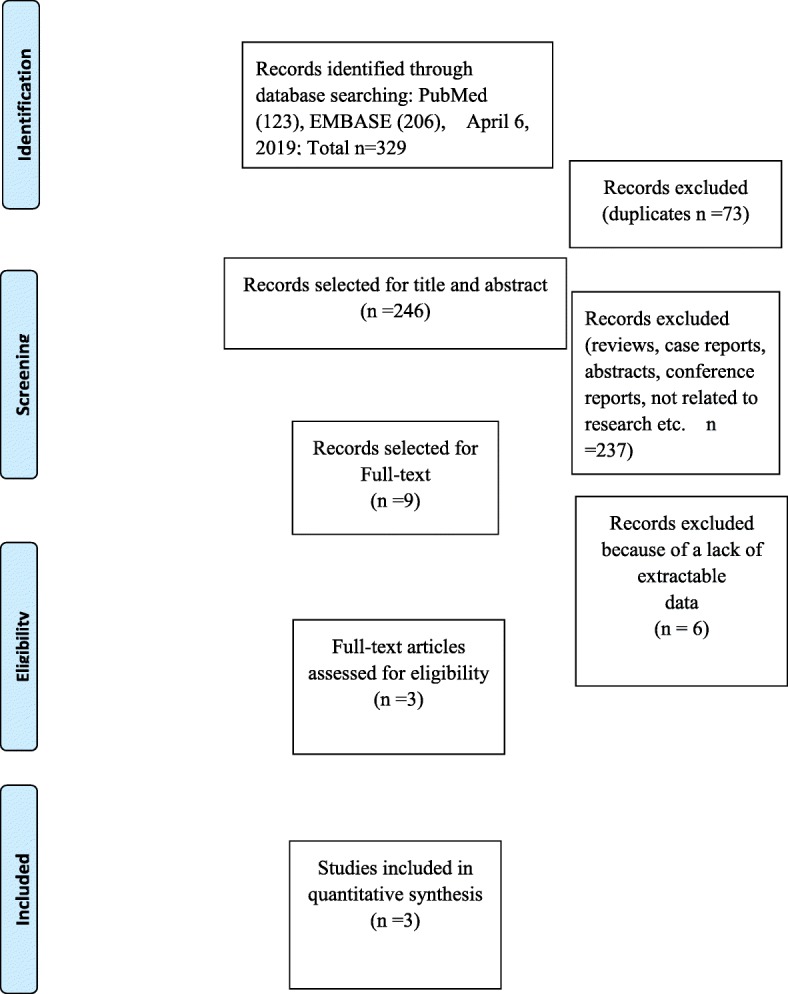
Flow chart illustrating the literature search process in this study

A total of 329 articles were initially identified from the PubMed and EMBASE databases. There were no additional studies from other sources. After removing the duplicate articles, 73 studies were included for further assessment. We reviewed the titles, abstracts, and full texts of all retrieved articles using the defined criteria. Finally, there were three articles that met the inclusion criteria. Figure 1 shows the flow diagram of the selection process. The characteristics of the included studies are shown in Table 1. All three articles were retrospective case-control studies from China [13–15].

**Table 1:** Characteristics of the three retrospective cohort studies

Author, year, location	Study design	Number of patients		Operative time (min)		bleeding volume (ml)		VAS score		ODI score		Local vertebral Cobb angle		Cement leakage	
		PKP	SSF + VP	PKP	SSF + VP	PKP	SSF + VP	PKP	SSF + VP	PKP	SSF + VP	PKP	SSF + VP	PKP	SSF + VP
Liang Chen, 2015, China	Retrospective cohort	31	23	76 (60–95)	128 (95–165)	Minimal	245	Pre7.8 ± 0.9 POM 3.3 ± 1.0 Fin 2.9 ± 0.9	Pre7.2 ± 1.6 POM 4.7 ± 1.3 Fin 3.5 ± 1.2	ND	ND	Pre 22.7 ± 6.9 POM 14.5 ± 5.3 Fin 15.5 ± 5.2	Pre 24.7 ± 9.2 POM 15.5 ± 6.2 Fin 15.8 ± 5.6	11	5
Yan-Sheng Huang, 2018, China	Retrospective cohort	32	28	43.1 ± 7.1	115.9 ± 10.0	9.9 ± 2.7	214.3 ± 17.5	Pre 8.1 ± 0.8 POM 2.8 ± 0.8 Fin 2.9 ± 1.2	Pre 8.0 ± 0.9 POM 3.8 ± 0.7 Fin 2.7 ± 1.3	Pre 75.3 ± 5.0 Fin 34.4 ± 5.0	Pre 74.4 ± 5.1 Fin 33.1 ± 4.4	Pre 22.9 ± 3.9 POM 14.5 ± 3.8 Fin 15.1 ± 4.6	Pre 22.6 ± 5.9 POM 13.6 ± 3.9 Fin 14.0 ± 4.4	3	3
Hou-Kun Li, 2017, China	Retrospective cohort	25	21	43.2 ± 21.8	230.6 ± 87.1	5.3 ± 3.1	215.0 ± 170.2	Pre 8.1 ± 0.8; POM 2.8 ± 0.8 Fin 2.9 ± 1.2	Pre 7.0 ± 1.4 POM 1.6 ± 0.9 Fin 1.2 ± 0.9	Pre 72.5 ± 10.0 Fin 27.2 ± 9.0	Pre 77.5 ± 10.6 Fin 26.0 ± 6.3	Pre 22.8 ± 7.4 POM 14.9 ± 8.2 Fin 17.0 ± 7.2	Pre 21.7 ± 3.6 POM 15.0 ± 6.7 Fin 16.5 ± 2.8	2	1

*Abbreviations*: *PKP* percutaneous kyphoplasty, *SSF* short-segmental fixation, *VP* vertebroplasty, *VAS* visual analog scale, *ODI* Oswestry Disability Index, *Pre* preoperative, *POM* postoperative 1 month, *Fin* final follow-up, *ND* no data

### Analysis

**Fig. 2:**
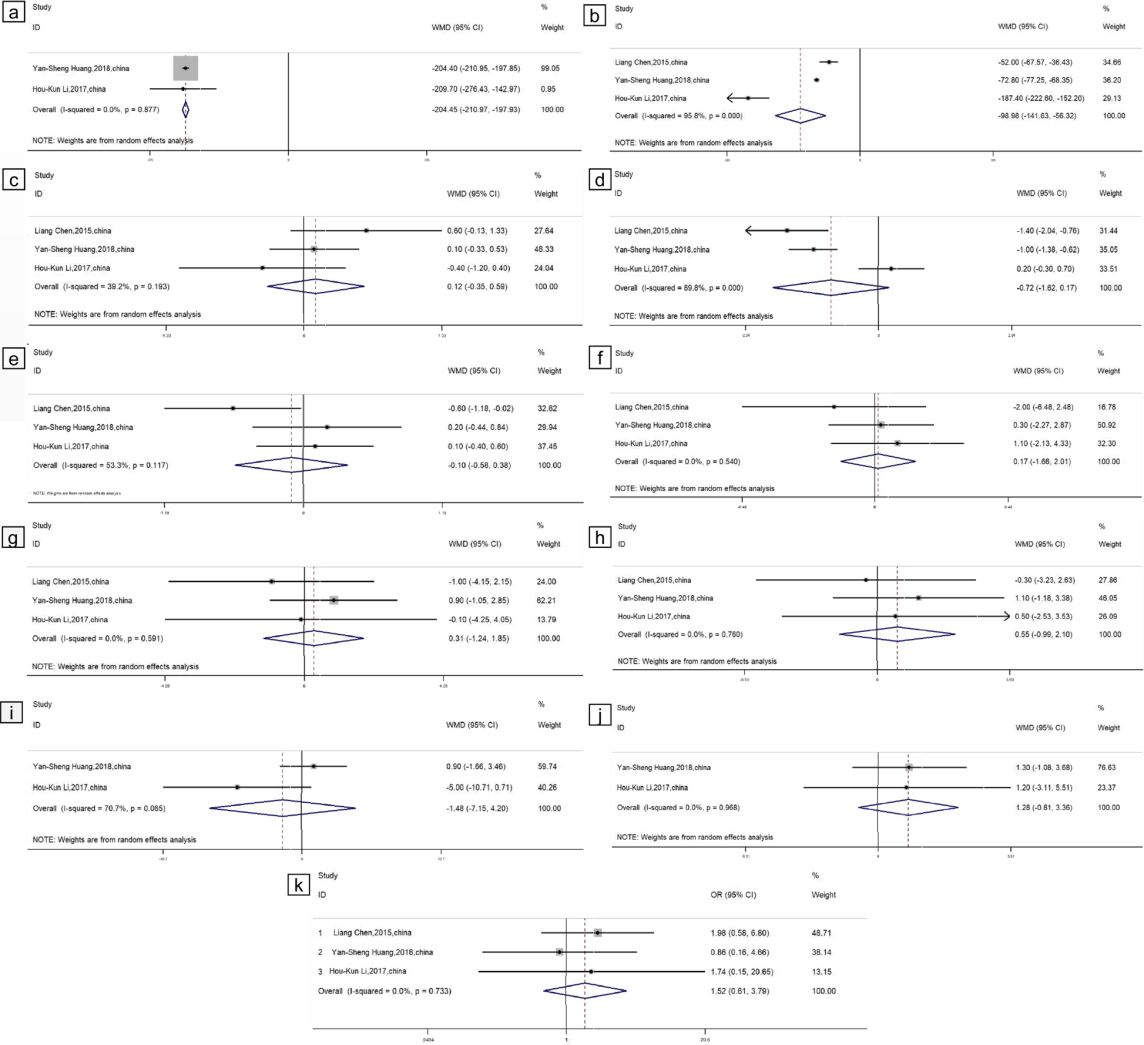
Forest plots comparing the outcome indicators of PKP and SSF + VP. **a** Bleeding volume. **b** Operation time. **c** Preoperative VAS score. **d** 1 month postoperative VAS score. **e** Final follow-up VAS score. **f** Preoperative local vertebral Cobb angle. **g** Postoperative 1 month local vertebral Cobb angle. **h** Final follow-up local vertebral Cobb angle. **i** Preoperative ODI score. **j** Postoperative final follow-up ODI score. **k** Cement leakage

The data of the three included articles were summarized and analyzed. Two indicators obtained from the forest map were statistically significant, namely, the volume of blood loss during surgery and the operation time. Since the specific volume of blood loss was not provided in one paper (Chen, China) [14], we combined the data of the other two papers, as shown in Fig. 2a. The combined weighted mean difference (WMD) (95% CI) was − 0.204.46 (− 210.97, − 197.93), and the combined results were statistically significant.

The operation time data of the three references were combined. As shown in Fig. 2b, the combined WMD (95% CI) was − 98.98 (− 141.63, − 56.32), and the combined results were statistically significant.

Data from the three included studies regarding the VAS score, ODI score, and local vertebral Cobb angle of the two different surgical methods PKP and SSF + VP were extracted and combined. We combined the VAS score data in the preoperative period, at 1 month postoperative and at the final follow-up. As shown in Fig. 2c–e, the combined WMDs (95% CI) were 0.12 (− 0.35, 0.58), 0.72 (− 1.62, 0.17), and − 0.10 (− 0.58, 0.38) in the preoperative period, at 1 month postoperative and at the final follow-up, respectively. The combined data showed no statistical significance.

We combined the local vertebral Cobb angle data in the preoperative period, at 1 month postoperative, and at the final follow-up, as shown in Fig. 2f–h. The corresponding WMDs (95% CI) were 0.17 (− 1.66, 2.01), 0.31 (− 1.24, 1.85), and 0.55 (− 0.99, 2.10); the combined data were not statistically significant.

Because (Chen, China) [14] did not provide ODI scores, the ODI scores in the preoperative period and at the postoperative final follow-up of the two remaining papers were combined, as shown in Fig. 2i, j. The WMDs (95% CI) in the preoperative period and at the postoperative final follow-up were − 1.48 (− 7.25, 4.20) and 1.28 (− 0.81, 3.36), respectively; the combined data were not statistically significant.

The cement leakage data were combined, as shown in Fig. 2k, with an OR (95% CI) value of 1.52 (0.61, 3.79); the combined data were not statistically significant.

## Discussion

In the past, KD was considered a rare disease. However, with the development of osteoporosis research and medical imaging technology, the diagnostic rate of osteoporotic vertebral fracture has increased in recent years. KD cannot be treated by conservative treatment such as lying in bed or with a brace [16]. KD is progressive and thus, self-healing is difficult. KD can cause long-term low back pain. If the fracture is not treated in time, the collapsed vertebral body can also compress the spinal cord and can even cause paralysis [2, 17, 18]. Compared with PVP, PKP has advantages in correcting kyphosis and reducing bone cement leakage. PKP has been widely used to treat KD patients without neurological symptoms. However, the special pathological changes of KD lead to poor dispersion of the bone cement through the cancellous bone, resulting in reduced mechanical stability. Cases of bone cement displacement, loss of vertebral body height, and even secondary paralysis have been reported in the literature [7–10]. Recently, short-segmental fixation combined with vertebroplasty (SSF + VP) has emerged as another option for KD treatment [1, 11, 12].

To the best of our knowledge, this analysis is the first to evaluate the efficacy of these two methods and provide evidence-based medical support for the decision-making process when treating KD patients without neurological deficits. Our study included three retrospective case-control studies [13–15]. Because one paper (Chen, China) [14] did not provide ODI scores or bleeding volume data, we included only the ODI score and bleeding volume data from the remaining two articles [13, 15]. The included operation time, VAS score, local vertebral Cobb angle, and cement leakage data were from all three studies [13–15].

According to our data analysis results, the combined data showed that the VAS score, ODI score, local vertebral body Cobb angle, and cement leakage were not significantly different between the two methods. The two surgical methods had a similar effect on improving the VAS score and ODI score and correcting the local vertebral body Cobb angle; the incidence of cement leakage was similar for both methods.

At present, the optimal treatment strategy is still controversial; however, the main purpose of both the PKP and SSF + VP surgical treatments is to relieve pain, restore vertebral height, and correct kyphosis. To improve treatment efficacy and patient satisfaction, the most important aspect is to relieve pain associated with KD. Pain is mainly due to the micromovements of the vertebral fracture. Therefore, eliminating the microfractures to achieve vertebral stabilization is very important. As a filler, bone cement provides immediate stability through microinterlock, volume-filling and bulk-filling mechanisms [19]. KD patients do not have obvious early symptoms, but these patients usually experience severe back pain and kyphosis by the time of diagnosis. Moreover, unlike common osteoporotic vertebral compression fractures (OVCFs), KD is characterized by sclerosis and bone resorption. Thus, the type of cement filling applied for treatment is very special. For most patients, bone cement is used as a support block because the bone cement does not diffuse sufficiently into the surrounding cancellous bone. Therefore, the anchoring effect between cement and bone is insufficient. In theory, SSF + VP will provide a more effective fixation than PKP and therefore will also lead to better pain relief, but this is not the case. The reason may be because PKP provides sufficient stability to the vertebra and destroys the surrounding pain sensory nerves [19, 20].

Bone cement leakage is a common complication and affects the patient’s surgical safety outcomes and prognosis [21]. The incidence of bone cement leakage is significantly increased for KD patients [22–24].

Some studies [11, 12, 20] believe that with the SSF + VP method, the bone cement can be injected under direct visualization after vertebral body reduction, which not only leads to a low incidence of bone cement leakage but also achieves a favorable vertebral height recovery. However, there were no significant differences in our study, most likely because the sample size was small.

In contrast, the combined data showed that the differences in bleeding volume and operation time were statistically significant. Relative to SSF + VP, PKP can shorten the surgical time and reduce the amount of bleeding. Moreover, Formica et al. [25] proposed a classification based on imaging findings that combines local and global sagittal parameters to help personalize the diagnosis and treatment of KD. Some studies [26, 27] suggest that vertebroplasty combined with posterior fixation can achieve a better sagittal deformity correction. KD usually occurs in adults over the age of 50, so the elderly patients in this study may have multiple comorbidities and severe osteoporosis; for such patients, the goals of the surgery should be to ensure safety and efficacy, minimize trauma, reduce bleeding, and shorten the operation time. These goals will maximize the benefits to the patient, thereby reducing the so-called domino effect of new fractures in adjacent segments due to poor sagittal alignment. Therefore, we believe that clinically, the decision to adopt SSF + VP should be made cautiously, and the decision requires a comprehensive analysis of each specific case.

In conclusion, both the PKP and SSF + VP methods are safe and effective for treating KD patients without neurological symptoms. However, PKP can shorten the operation time and reduce the bleeding volume. However, due to the small number of randomized controlled studies included in this systematic evaluation, more prospective randomized controlled studies are needed to strengthen the credibility of this result. In addition, this study also has the following limitations: first, there was some statistical heterogeneity between the included studies. Although we used the random-effects model to balance this statistical heterogeneity in the analysis process, this heterogeneity still has some influence on the conclusions. Second, all the included studies were retrospective case-control studies, and this non-randomized controlled study was susceptible to selection bias, implementation bias, mixed bias, etc.

## Data Availability

All data analyzed during this study are included in this published article.

## Abbreviations

CT: Computed tomography
Fin: Final follow-up
KD: Kummell disease
MRI: Magnetic resonance imaging
ND: No data
ODI: Oswestry Disability Index
PKP: Percutaneous kyphoplasty
POM: Postoperative 1 month
Pre: Preoperative
PVP: Percutaneous vertebroplasty
RCTs: Randomized controlled trials
SSF: Short-segmental fixation
VAS: Visual analog score
VP: Vertebroplasty
WMDs: Weighted mean differences
